# The effects of same-session combined exercise training on cardiorespiratory and functional fitness in older adults: a systematic review and meta-analysis

**DOI:** 10.1007/s40520-019-01124-7

**Published:** 2019-01-19

**Authors:** Christopher Hurst, Kathryn L. Weston, Shaun J. McLaren, Matthew Weston

**Affiliations:** 1 AGE Research Group, Institute of Neuroscience, Newcastle University, Campus for Ageing and Vitality, 1st Floor Biomedical Research Building, Newcastle upon Tyne, NE4 5PL UK; 2 NIHR Newcastle Biomedical Research Centre, Newcastle University and Newcastle upon Tyne Hospitals NHS Foundation Trust, Newcastle upon Tyne, UK; 3 Newcastle University Institute for Ageing, Newcastle upon Tyne, UK; 4 School of Health and Social Care, Teesside University, Middlesbrough, UK; 5 Institute for Sport, Physical Activity and Leisure, Leeds Beckett University, Leeds, UK; 6 The Rugby Football League, Leeds, UK

**Keywords:** Ageing, Combined training, Endurance training, Strength training, Physical fitness

## Abstract

Endurance and strength training are effective strategies for counteracting age-associated reductions in physical performance in older adults, with a combination of both exercise modes recommended to maximise potential fitness benefits. This meta-analysis sought to quantify the effects of same-session combined endurance and strength training on fitness in adults aged over 50 years. Five electronic databases were searched with studies required to include one of the following outcome measures: *V*O_2peak_, 6-min walk test (6MWT), 8-ft timed up-and-go (TUG), and 30-s chair stand. Separate random-effects meta-analyses compared combined training with (1) no-exercise control, (2) endurance training, and (3) strength training with probabilistic magnitude-based inferences subsequently applied. Twenty-seven studies involving 1346 subjects with a mean age of 68.8 years (range 54–85 years) were included in the analysis. The meta-analysed effect on *V*O_2peak_ was a moderately beneficial effect for the combined training compared to no-exercise controls (3.6 mL kg^−1^ min^−1^; ± 95% confidence limits 0.8 mL kg^−1^ min^−1^) with additional increases for studies with greater proportions of female participants and shorter training interventions. Combined training also had small-to-moderately beneficial effects on *V*O_2peak_ when compared to endurance training (0.8 mL kg^−1^ min^−1^; ± 1.0 mL kg^−1^ min^−1^), 30-s chair stand when compared with strength training (1.1 repetitions; ± 0.5 repetitions) and on TUG (0.8 s; ± 0.7 s), 30-s chair stand (2.8 repetitions; ± 1.7 repetitions), and 6MWT (31.5 m; ± 22.4 m) when compared to no-exercise controls. All other comparisons were unclear. Same-session combined training can induce clinically relevant fitness improvements in older adults.

**Electronic supplementary material:**

The online version of this article (10.1007/s40520-019-01124-7) contains supplementary material, which is available to authorized users.

## Introduction

Human ageing is associated with progressive declines across multiple physiological systems, with changes in the cardiorespiratory and neuromuscular systems some of the most pronounced. Reduced levels of cardiorespiratory [1] and muscular fitness [2] have been associated with increased mortality and morbidity. Improving both of these physical components offers the most effective strategy to reduce all-cause and cardiovascular mortality risk [3]. However, despite the implications of reduced physiological functioning on disease risk and lifespan, maintaining an independent and inclusive lifestyle may be of greater relevance to older adults. Previous work has demonstrated that cardiorespiratory fitness is related to functional capacity and the odds of independent living [4, 5], while muscular fitness (e.g., muscular strength and power) is a critical determinant of physical functioning in older adults [6, 7]. Despite the inevitable declines in physiological functioning observed with ageing, older adults remain highly trainable into advanced age with substantial fitness improvements possible following short-term training programmes [8, 9]. As higher levels of both cardiorespiratory and muscular fitness are related to improved functional performance and reduced mortality risk, these physical components are key targets for intervention.

Traditionally, endurance-type activities (e.g., running, cycling) are prescribed for improving cardiorespiratory fitness with muscle-strengthening activities (e.g., free weights, resistance machines, elastic resistance bands) used to improve muscular fitness. However, as neither endurance nor strength training performed in isolation promotes holistic fitness improvement, exercise recommendations for older adults typically advocate training programmes consisting of a combination of endurance and strength training activities [10, 11]. As a result, ‘combined’ or ‘concurrent’ training programmes—involving endurance and strength training performed within the same, or separate exercise sessions of a training programme, respectively—are commonly prescribed. This approach has been suggested to be a more effective strategy than either endurance or strength training performed alone because of the potential to impact upon multiple components of fitness simultaneously [10, 12]. Recent observational data support this assertion as older adults who meet both the endurance and muscle-strengthening activity guidelines perform significantly better on measures of muscular and functional fitness [13].

However, despite the well-documented and wide-ranging benefits of exercise training, the requirement to perform separate endurance and strength training sessions places considerable time demands on individuals. This remains an important consideration in a population where adherence to exercise guidelines remains poor [14] and where lack of time remains one of the most commonly cited barriers to exercise [15]. Consequently, training programmes involving a reduced time commitment via delivery of endurance and strength training within the same training session may be a more time-efficient, and thereby, attractive proposition for potential exercisers. It seems feasible to suggest that asking individuals to complete a reduced frequency of exercise sessions per week may be more achievable.

Although previous work has reviewed strategies and provided recommendations for the prescription of combined exercise training in older adults [12], there is currently no systematic review examining the effect of same-session combined exercise training on measures of fitness in older adults. Accordingly, the aim of our investigation was to systematically review and meta-analyse the effects of same-session combined exercise training on measures of fitness in adults aged over 50 years, while also exploring the modifying effects of study and subject characteristics. By doing so, we aimed to provide a practically relevant quantification of this training approach to support clinicians and practitioners to make informed decisions relating to the prescription of exercise training.

## Methods

### Protocol and registration

This review was carried out in accordance with the Preferred Reporting Items for Systematic Reviews and Meta-Analyses (PRISMA) guidelines [16] and was prospectively registered on the International Prospective Register of Systematic Reviews (PROSPERO) as CRD42015019577.

### Search strategy

Electronic searching of five databases (PubMed, MEDLINE, Scopus, BIOSIS, and Web of Science) was performed from the earliest date available up to July 2018. Independent variable search terms were ‘multicomponent training’, ‘multicomponent exercise’, ‘circuit training’, ‘circuit resistance training’, ‘combined exercise training’, ‘combined training’, ‘multi-modal exercise training’, ‘same-session exercise’, ‘concurrent training’, and ‘concurrent exercise’. Dependent variable search terms were ‘functional fitness’, ‘functional performance’, ‘physical performance’, ‘quality of life’, ‘functional decline’, ‘aerobic fitness’, ‘strength’, and ‘power’. Independent variable search terms were combined with dependent variable search terms using the ‘AND’ operator, giving a total of 80 search combinations. Reference lists from retrieved studies were also examined for potentially eligible papers.

### Inclusion criteria

#### Study design

This review considered only original research articles, published in English. Randomised and non-randomised controlled trials were included, while uncontrolled, cross-sectional, and single-group, pre–post studies were excluded.

#### Participants

Only studies involving healthy, community-dwelling participants aged > 50 years were included. We defined an age of 50 years as our cut-off point for inclusion as there are clear physical and physiological declines beyond this age in adults [17, 18]. In addition, evidence suggests that physical capability in midlife is predictive of physical performance in later life [19], implying that earlier intervention at ~ 50 years of age can have positive long-term implications. It is acknowledged, however, that the use of an arbitrary threshold implies synonymy between chronological and biological age; yet, there remains no consensus on when old age begins, suggesting that any arbitrary definition is likely to be imperfect [20].

Studies involving participants with non-communicable disease (e.g., cardiovascular disease, type 2 diabetes mellitus, cancers, and chronic obstructive pulmonary disease) or who were being prescribed a specific pharmacological treatment were excluded. Studies were not automatically excluded if participants were labelled as an alternative population group (e.g. obese), as these were considered an extension of a healthy population rather than an alternative clinical group. For example, the study of Stewart et al. [21] categorised their participants as having ‘untreated milder forms of hypertension’, so was suitable for inclusion.

#### Training interventions

To be considered for inclusion in this systematic review, studies were required to include at least one combined exercise training group and a comparator group of either (1) no-exercise control; (2) endurance training only; or (3) strength training only. To be considered ‘combined training’ each training session within the intervention had to contain discrete, standalone activities of (1) endurance training and (2) strength training. Endurance training was defined as exercise involving large muscle groups in dynamic activities that result in substantial increases in heart rate and energy expenditure and was not limited to any specific modes of exercise [22]. Strength training was defined as any muscle-strengthening activities including where participants worked against or moved an external resistance (e.g., free weights, weight machines, elastic resistance bands, body weight exercises) [22, 23]. Interventions where training sessions contained additional training elements targeting improvement in other fitness components (e.g., balance, flexibility, coordination) were not excluded if endurance and strength training activities were present in each session. Activities prescribed as ‘warm up’ or ‘recovery’ were not considered. Training interventions were required to be a minimum of 2 weeks in duration with all training sessions supervised to ensure the fidelity of the intervention as previous work has suggested that exercise adherence is generally higher in supervised programmes [24] and observed effects may be greater when training is supervised [25]. Studies involving nutritional interventions were only included if there was a combined training and a comparator group (described previously) which were not exposed to these interventions. Interventions labelled as ‘circuit training’ typically involving subjects performing resistance exercises interspersed with aerobic exercises were not included as circuit training and combined training are two discrete training modes.

#### Outcome measures

As an important aim of the present work was to generate practically relevant information for clinicians and practitioners in the applied environment, we sought to provide meaningful context to our results by reporting raw mean differences rather than standardised mean differences (SMD). The SMD can be difficult to interpret on a practical level [26] and the use of the standard deviation (SD) to standardise each effect can introduce heterogeneity that is unrelated to any real differences in the effect between studies [27]. The SMD is often used when studies assess the same outcome but measure it in a variety of ways; however, it may be inappropriate to use different, albeit similar tests of physical fitness interchangeably unless equivalence has been demonstrated. This is because successful performance may be determined by different physiological parameters and issues such as reliability and test sensitivity may vary [28].

Accordingly, we selected a range of specific outcome measures to assess the effectiveness of same-session combined exercise training with our decision influenced by the personal experience of the authors as well as the desire to include functionally relevant measures of fitness with limited floor and ceiling effects. Studies required to contain at least one of the following outcome measures to be included in this meta-analysis: (1) peak oxygen uptake (*V*O_2peak_) or maximal oxygen uptake (*V*O_2max_), assessed via maximal incremental test—associated with the ability to maintain independent function and prevent disability [4, 5]; (2) six-minute walk test (6MWT)—a valid and reliable measure of physical endurance associated with self-reported functional ability with performance determined by leg strength and power [29, 30]; (3) 8-ft timed up-and-go (TUG)—a composite measure of performance related to dynamic balance and mobility measured over a distance of 8 ft [31]. We selected the 8-ft distance as previous authors [31] have suggested that this version of the test can be more feasibility administered in a home setting, is simpler for participants to perform, and has better sensitivity than alternative versions [28]; (4) 30-s chair stand—a valid measure of lower body muscle functioning [32] capable of detecting change in functional capacity in older adults [33]. Assessment of functional fitness (e.g., 30-s chair stand, TUG) provides a composite measure of physical capability as successful performance on these tests is determined by several factors [34], providing a functionally relevant and ecologically valid assessment of fitness.

### Study selection

**Fig. 1:**
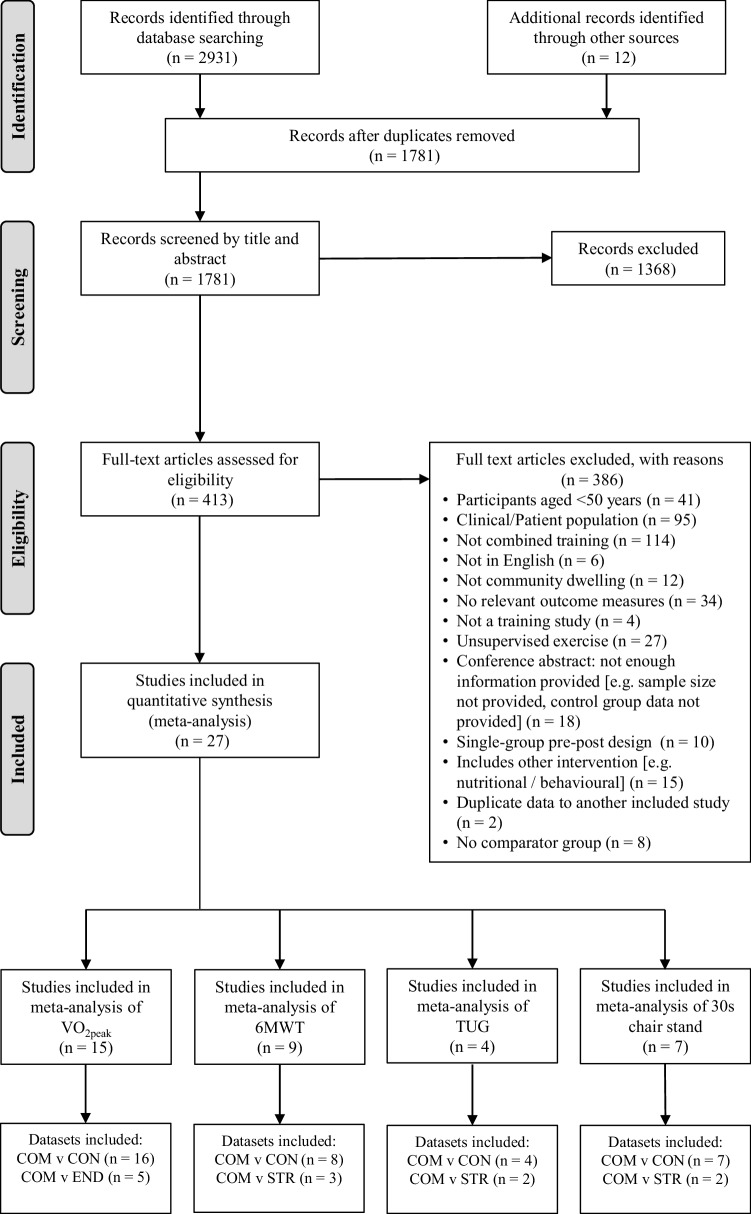
PRISMA flow diagram of the study selection process. *VO*_*2peak*_ peak oxygen uptake, *6WMT* 6-min walk test, *TUG* timed up and go, *COM* combined training, *CON* no-exercise control, *END* endurance training only, *STR* strength training only

To identify relevant studies, all records were screened independently for eligibility by two authors (CH and KLW) with any disagreements resolved by a third reviewer (MW). Papers that were clearly not relevant were removed from the database list before assessing all other titles and abstracts using our pre-determined inclusion and exclusion criteria. Following this, full-text papers, including reviews, were then collected for evaluation. When full texts were not available, the corresponding author was contacted. After removal of duplicates and elimination of papers based on title and abstract screening, there were 413 studies remaining (Fig. 1). After evaluation of full texts, there were 27 papers that met our inclusion criteria and were, therefore, included in the meta-analysis.

### Data extraction

Data were extracted from eligible studies into a custom-made spreadsheet by the lead author with a second investigator checking the extracted data for accuracy (KW/SM). Mean and standard deviation of pre-training and post-training values along with sample size from the combined and comparator groups were extracted for each outcome measure. In studies where standard deviations were not reported, they were calculated using the standard errors or confidence intervals provided [35]. Data for potential moderator variables that could reasonably influence the overall effect of training on changes in fitness were also extracted. This included both participant (mean age, proportion of males, and baseline fitness) and intervention characteristics [intervention duration (weeks) and training frequency (sessions per week)]. Where intervention duration was expressed as months, this value was converted to weeks based on 1 month being equal to 4.3 weeks. Attempts were made to contact corresponding authors via e-mail to obtain further information or clarity when needed.

In studies with multiple data sets [36–41] where more than one comparison was possible (e.g., combined versus no-exercise control and combined versus endurance only) sample size was halved where necessary to avoid double counting in analysis [42]. Several studies [36, 43–45] reported data at several time points or after a period of detraining; in all instances, pre- and post-intervention data only were analysed. In studies where there were more than one combined training group performing the same exercise training, but, in a different order (i.e., endurance training first or strength training first within a session) [37, 46, 47], data were combined using procedures as described in the Cochrane handbook [35].

### Assessment of study quality

Methodological risk of bias was assessed by two investigators (CH and KW) according to the Cochrane Collaboration’s tool for assessing risk of bias [35]. Any disagreements were resolved by discussion to reach consensus.

### Data analysis

#### Meta-analysis

All data analyses were performed using Comprehensive Meta-Analysis software, version 3 (Biostat Inc., Englewood, NJ, USA). Separate random-effects meta-analyses were performed to determine the pooled effect of change in each outcome measure for (1) combined training compared with no-exercise control, (2) combined training compared with endurance training only, and (3) combined training compared to strength training only. The precision of the pooled effect was expressed as 95% confidence limits (CL), calculated using the Knapp and Hartung approach [48]. To provide a real-world, measure for practical/clinical interpretation of our results, we evaluated the effects for each outcome measure against pre-specified thresholds for small, moderate, and large effects [49]. As robust clinical anchors for our outcome measures remain to be determined in this population, magnitude of effects were defined as standardised mean differences of 0.2, 0.6, and 1.2 between-subject standard deviations (SD) for small, moderate, and large effects, respectively [50]. The SD of the pooled baseline values was used for this purpose, as the post-intervention SD can be inflated by individual differences in response to an intervention [51]. Magnitude thresholds for small, moderate, and large effects, respectively, were: *V*O_2peak_, 0.6, 1.8, and 3.6 mL kg^−1^ min^−1^; 6MWT, 12.5, 37.6, and 75.1 m; TUG, 0.2, 0.5, and 1.1 s; 30 s-sit-to-stand, 1, 3, and 6 repetitions (expressed as an integer as partial repetitions are not possible).

Using the pooled effect for each outcome measure, together with its uncertainty (i.e. the confidence interval), the probability of the true effect being trivial, beneficial, or harmful was calculated, and then interpreted using the following scale: < 0.5%, most unlikely or almost certainly not; 0.5–5%, very unlikely; 5–25%, unlikely or probably not; 25–75%, possibly; 75–95%, likely; 95–99.5%, very likely; > 99.5%, most likely [50]. Effects were evaluated clinically, given that exercise interventions can be potentially harmful (i.e., reduce physical performance and functional capacity) as well as beneficial to individuals, and were considered unclear if the chance of benefit (improved physical performance) was high enough to warrant use of the intervention but with an unacceptable risk of harm (reduced physical performance). An odds ratio of benefit to harm of < 66 was used to identify such unclear effects. Between-study heterogeneity [Tau (*τ*)] was expressed as an SD [52], calculated using DerSimonian and Laird’s generalised method of moments [53], and doubled to interpret its magnitude against the above scale of effect sizes [54].

#### Meta-regression

Meta-regression was performed to explore the effect of five putative moderator variables which could reasonably influence the effect of training on fitness. These continuous variables were baseline fitness, intervention duration, weekly training frequency, age, and maleness (i.e., the proportion of males in the study sample). The modifying effects of these variables were calculated as the effect of two SDs (i.e., the difference between a typically low and a typically high value) and were evaluated non-clinically [50]. Meta-regression was performed only when there were > 10 data sets [35].

#### Publication bias

Publication bias was assessed using Egger’s test to evaluate asymmetry of funnel plots [55]. However, caution in interpreting these results is warranted when there are less than ten studies in the meta-analysis as the power of the test is too low to distinguish chance from real asymmetry [35].

## Results

### Study characteristics

Final analysis included 27 studies involving 1346 subjects with a mean age of 68.8 ± 5.9 years. There were 15 studies included in the meta-analysis of *V*O_2peak_ [21, 38–40, 46, 47, 56–64], nine studies for 6MWT [37, 41, 43–45, 65–68], four for TUG [41, 45, 65, 69], and seven for 30-s chair stand [36, 41, 45, 46, 65, 68, 70]. Overall, there were 24 studies which included a no-exercise control group, six studies including a strength training only group, and seven studies including an endurance training only group. Individual study characteristics and intervention characteristics can be found in Table 1 and the electronic supplementary material (ESM) available with this article (ESM 1).

**Table 1:** Descriptive characteristics of included studies

Study	Study design		Participants			Outcome measures
	Randomised or non-randomised	Experimental groups	Age (years)	*n* allocated (male/female)	*n* analysed (male/female)	
Cadore et al. [38]	Randomised	COM	66.8 ± 4.8	10 (10/0)	8 (8/0)	VO_2peak_
		STR	64.0 ± 3.5	10 (10/0)	8 (8/0)	
		END	64.4 ± 3.5	9 (9/0)	7 (7/0)	
Campos et al. [37]	Randomised	COM	62.0 ± 2.5	6 (0/6)	5 (0/5)	6MWT
		COM	66.0 ± 3.5	6 (0/6)	5 (0/5)	
		END	63.6 ± 2.5	6 (0/6)	5 (0/5)	
		STR	70.0 ± 6.3	6 (0/6)	4 (0/4)	
		CON	74.0 ± 4.4	6 (0/6)	3 (0/3)	
Carvalho et al. [45]	Randomised	COM	68.4 ± 2.9	32 (0/32)	32 (0/32)	6MWT, TUG, 30-s chair stand
		CON	69.6 ± 4.2	25 (0/25)	25 (0/25)	
Cress et al. [56]	Non-randomised	COM	71.1 ± 5.1	20 (0/20)	17 (0/17)	VO_2peak_
		CON	73.3 ± 6.7	11 (0/11)	10 (0/10)	
Cress et al. [59]	Randomised	COM	75.6 ± 3.6	23 (?)	23 (?)	VO_2peak_
		CON	76.0 ± 5.1	26 (?)	26 (?)	
Delecluse et al. [40]	Randomised	COM	63.8 ± 4.8	22 (22/0)	20 (20/0)	VO_2peak_
		COM	63.7 ± 6.0	22 (22/0)	21 (21/0)	
		END	64.5 ± 5.3	21 (22/0)	21 (21/0)	
		CON	61.5 ± 5.0	13 (13/0)	13 (13/0)	
Desjardins-Crépeau et al. [68]	Randomised	COM	70.9 ± 7.4	?	16 (8/8)	6MWT, 30-s chair stand
		CON	72.5 ± 7.0	?	18 (15/3)	
Douda et al. [36]	Non-randomised	COM	65.6 ± 4.9	15 (0/15)	10 (0/10)	30-s chair stand
		END	63.8 ± 5.6	15 (0/15)	12 (0/12)	
		STR	62.1 ± 4.1	15 (0/15)	10 (0/10)	
		CON	66.2 ± 5.1	18 (0/18)	10 (0/10)	
Engels et al. [47]	Randomised	COM	68.6 ± 5.6^a^	12 (?)	10 (2/8)	VO_2peak_
		COM	68.6 ± 5.6 ^a^	11 (?)	10 (0/10)	
		CON	68.6 ± 5.6 ^a^	11 (2/9)	11 (2/9)	
Ferketich et al. [39]	Randomised	COM	67.2 ± 1.5	8 (0/8)	7 (0/7)	VO_2peak_
		END	69.2 ± 1.7	8 (0/8)	8 (0/8)	
		CON	69.8 ± 2.0	8 (0/8)	6 (0/6)	
García-Pinillos et al. [70]	Randomised	COM	73.5 ± 5.6	47 (13/34)	47 (13/34)	30-s chair stand
		CON	72.1 ± 5.8	47 (?)	43 (13/30)	
Kim et al. [41]	Randomised	COM	73.2 ± 4.9	16 (?)	13 (?)	6MWT, TUG, 30-s chair stand
		STR	73.2 ± 4.9	16 (?)	12 (?)	
		CON	73.2 ± 4.9	15 (?)	10 (?)	
King et al. [43]	Randomised	COM	77.0 ± 4.6	80 (18/62)	67 (?)	6MWT
		CON	77.9 ± 4.4	75 (15/60)	57 (?)	
Kwon et al. [62]	Non-randomised	COM	77.4 ± 2.6	20 (0/20)	20 (0/20)	VO_2peak_
		CON	77.0 ± 3.3	20 (0/20)	20 (0/20)	
Marques et al. [67]	Randomised	COM	68.6 ± 3.4	38 (0/38)	36 (0/38)	6MWT
		STR	68.1 ± 4.3	39 (0/39)	38 (0/38)	
Marques et al. [65]	Randomised	COM	70.1 ± 5.4	30 (0/30)	27 (0/27)	6MWT, TUG, 30-s chair stand
		CON	68.2 ± 5.7	30 (0/30)	22 (0/22)	
Park et al. [60]	Randomised	COM	68.3 ± 3.6	25 (0/25)	25 (0/25)	VO_2peak_
		CON	68.4 ± 3.4	25 (0/25)	25 (0/25)	
Park et al. [61]	Randomised	COM	66.1 ± 3.1	10 (0/10)	10 (0/10)	VO_2peak_
		CON	67.7 ± 5.2	10 (0/10)	10 (0/10)	
Park et al. [63]	Randomised	COM	57.2 ± 2.6	10 (0/10)	10 (0/10)	VO_2peak_
		CON	57.2 ± 1.7	10 (0/10)	10 (0/10)	
Puggaard [57]	Randomised	COM	65.0 (?)	12 (0/12)	12 (0/12)	VO_2peak_
		COM	75.0 (?)	12 (0/12)	12 (0/12)	
		COM	85.0 (?)	17 (0/17)	17 (0/17)	
		CON	65.0 (?)	2 (0/2)	2 (0/2)	
		CON	75.0 (?)	23 (0/23)	23 (0/23)	
		CON	85.0 (?)	16 (0/16)	16 (0/16)	
Rubenstein et al. [66]	Randomised	COM	76.4 ± 4.9	31 (31/0)	28 (28/0)	6MWT
		CON	74.4 ± 4.4	28 (28/0)	27 (27/0)	
Schaun et al. [64]	Randomised	COM	54.0 ± 4^a^	10 (10/0)	10 (10/0)	VO_2peak_
		END	54.0 ± 4 ^a^	10 (10/0)	10 (10/0)	
Stewart et al. [21]	Randomised	COM	63.0 ± 5.5	57 (?)	51 (25/26)	VO_2peak_
		CON	64.1 ± 3.1	58 (?)	53 (26/27)	
Timmons et al. [69]	Randomised	COM	69.2 ± 2.7	21 (16/5)	21 (16/5)	TUG
		CON	69.0 ± 3.3	21(8/13)	21(8/13)	
		END	69.2 ± 3.1	21 (11/10)	21 (11/10)	
		STR	69.6 ± 4.9	23 (?)	21 (10/11)	
Villareal et al. [58]	Randomised	COM	70.0 ± 4	26 (10/16)	26 (10/16)	VO_2peak_
		CON	69.0 ± 4	27 (9/18)	27 (9/18)	
Wang et al. [44]	Non-randomised	COM	70.3 ± 4.6	18 (?)	17 (4/13)	6MWT
		CON	70.5 ± 5.5	15 (?)	12 (4/8)	
Wilhelm et al. [46]	Non-randomised	COM	67.1 ± 6.1	15 (15/0)	12 (12/0)	VO_2peak_, 30-s chair stand
		COM	63.2 ± 3.3	15 (15/0)	11 (11/0)	
		CON	65.8 ± 5.3	15 (15/0)	13 (13/0)	

Data are presented as mean ± standard deviation unless otherwise stated

*COM* combined training, *END* endurance training only, *STR* strength training only, *CON* no-exercise control group, *VO*_*2peak*_ peak oxygen uptake, *6MWT* 6-min walk test, *TUG* timed up-and-go, *?* data unknown/not presented by authors

^a^Age not reported per group

### Risk of bias assessment

**Fig. 2:**
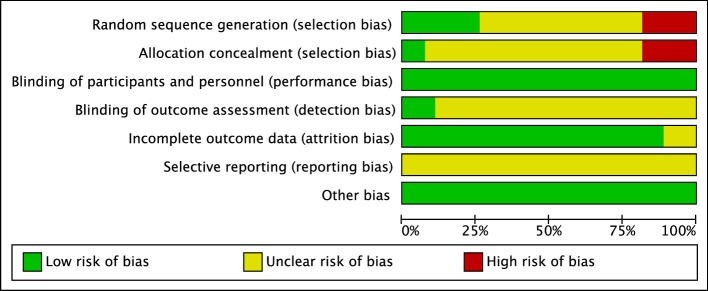
Risk of bias summary

A summary of risk of bias assessment is presented in Fig. 2. Individual study-level data can be found in the electronic supplementary material available with this article (ESM 2). Risk of bias was predominantly low or unclear across all domains.

### Effect of combined exercise training on *V*O_2peak_

**Fig. 3:**
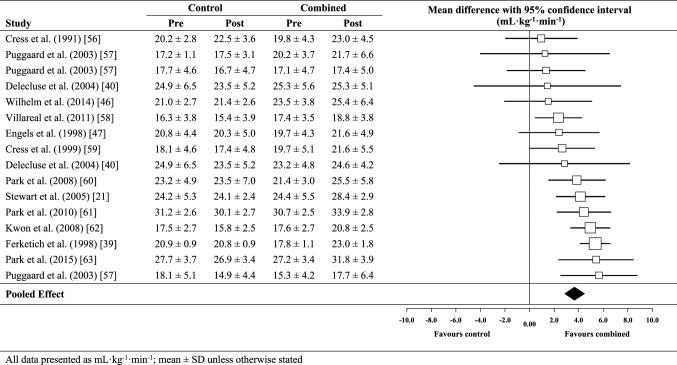
Individual study data and meta-analysed effect of combined training versus no-exercise control on peak oxygen uptake (*V*O_2peak_)

**Fig. 4:**
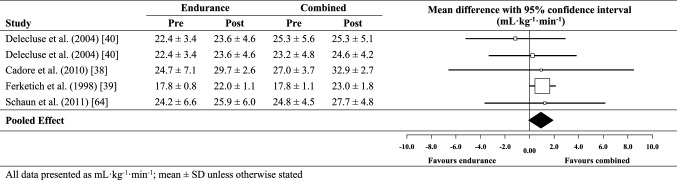
Individual study data and meta-analysed effect of combined training versus endurance training on peak oxygen uptake (*V*O_2peak_)

The meta-analysed effect of combined training, when compared to no-exercise controls (Fig. 3), was a most likely moderate (possibly large) beneficial effect on *V*O_2peak_ (3.6 mL kg^−1^ min^−1^; ± 95% confidence limits 0.8 mL kg^−1^ min^−1^). Between-study heterogeneity (*τ*) was ± 0.9 mL kg^−1^ min^−1^ (small magnitude). Egger’s coefficient was − 1.89 (95% CI − 3.27 to − 0.51; *p* = 0.01). Of the five moderator variables selected, meta-regression analysis revealed a greater additional beneficial effect (possibly moderate) for studies with a higher proportion of female participants (2.1 mL kg^−1^ min^−1^; ± 1.8 mL kg^−1^ min^−1^) and a likely small additional benefit for typically shorter training programmes (1.6 mL kg^−1^ min^−1^; ± 1.5 mL kg^−1^ min^−1^). The effect of all other putative modifiers was unclear. When compared against endurance training only (Fig. 4), the meta-analysed effect of combined training was a possibly small beneficial effect on *V*O_2peak_ (0.8 mL kg^−1^ min^−1^; ± 1.0 mL kg^−1^ min^−1^). Between-study heterogeneity was trivial (± 0 mL kg^−1^ min^−1^) and Egger’s coefficient was − 0.41 (− 1.62 to 0.80; *p* = 0.36).

### Effect of combined training on 6MWT performance

**Fig. 5:**
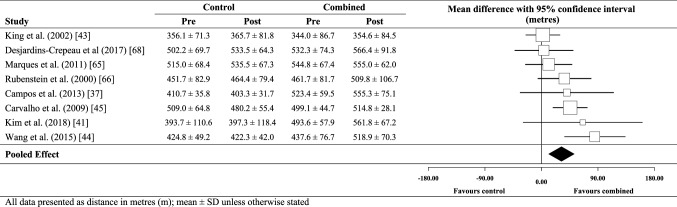
Individual study data and meta-analysed effect of combined training versus no-exercise control on 6MWT performance

**Fig. 6:**
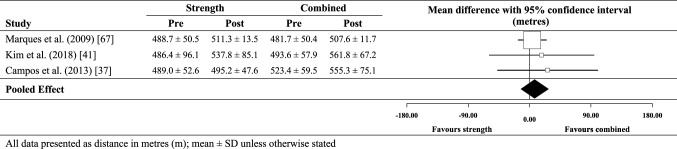
Individual study data and meta-analysed effect of combined training versus strength training on 6MWT performance

When compared to no-exercise controls (Fig. 5), there was a very likely small beneficial effect for combined training on 6MWT performance (29.6 m; ± 20.5 m). Between-study heterogeneity (*τ*) was small (± 18.7 m), while Egger’s coefficient was 1.26 (− 2.10 to 4.63; *p* = 0.39). The meta-analysed effect of combined training versus strength training only (Fig. 6) was unclear (5.8 m; ± 20.2 m) with between-study heterogeneity trivial (± 0 m) and Egger’s coefficient 0.65 (− 1.82 to 3.12; *p* = 0.18).

### Effect of combined training on TUG performance

**Fig. 7:**
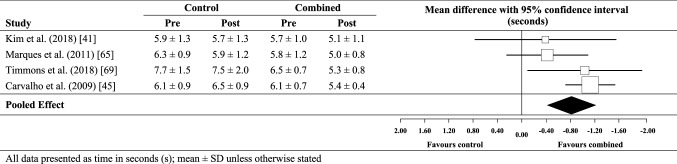
Individual study data and meta-analysed effect of combined training versus no-exercise control on TUG performance

**Fig. 8:**
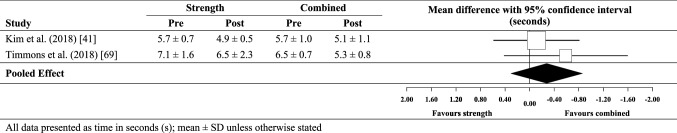
Individual study data and meta-analysed effect of combined training versus strength training on TUG performance

The meta-analysed effect of combined training compared with no-exercise controls (Fig. 7) was a likely moderate beneficial effect on timed up-and-go performance (0.8 s; ±0.4 s). Between-study heterogeneity (*τ*) was small (± 0.2 s), while Egger’s coefficient was − 1.63 (− 7.73 to 4.47); *p* = 0.37. Compared with strength training only (Fig. 8), the effect for combined training was unclear (0.3 s; ± 0.6 s). Assessment of publication bias was not possible for combined training versus strength training only as there were only two data sets. Between-study heterogeneity (*τ*) was trivial (± 0 s).

### Effect of combined training on 30-s chair stand test performance

**Fig. 9:**
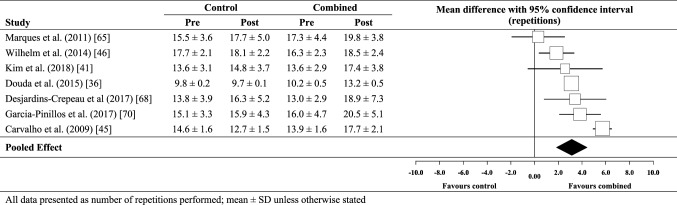
Individual study data and meta-analysed effect of combined training versus no-exercise control on 30-s chair stand performance

**Fig. 10:**
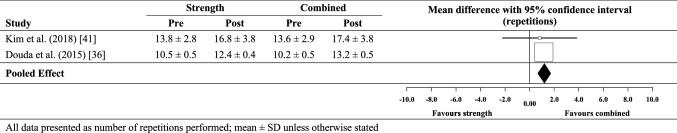
Individual study data and meta-analysed effect of combined training versus strength training on 30-s chair stand performance

Compared to no-exercise controls, the meta-analysed effect of combined training (Fig. 9) was a most likely small (possibly moderate) beneficial effect on 30-s chair stand performance (3.1 repetitions; ± 1.3 repetitions). Between-study heterogeneity (*τ*) was moderate (± 1.5 repetitions) and Egger’s coefficient was − 0.17 (− 4.37 to 4.04; *p* = 0.92). Compared with strength training only (Fig. 10), there was a possibly small beneficial effect for combined training (1.1; ± 0.5 repetitions). For combined training versus strength training only, there were only two data sets, so assessment of publication bias was not possible. Between-study heterogeneity (*τ*) was trivial (± 0 repetitions).

## Discussion

Given the proposed benefit of training programmes involving a combination of endurance and strength training on physical performance in older adults, we sought to systematically review and quantify the effects of same-session combined training on measures of fitness in adults aged over 50 years. Our results demonstrate clear and functionally relevant beneficial effects for combined training on *V*O_2peak_, 6MWT, TUG, and 30-s chair stand when compared with no-exercise controls. In addition, there was a small beneficial effect for combined training when compared to endurance training only for *V*O_2peak_.

The data reported in this investigation confirm the findings of previous experimental studies [21, 38, 39], demonstrating that combined training—advocated as a method for simultaneously improving cardiorespiratory and muscular fitness [12]—is an effective training strategy for improving *V*O_2peak_. The meta-analysed effect reported here is comparable to previous meta-analyses reporting mean improvements of 3.78 mL kg^−1^ min^−1^ [71] and 3.5 mL kg^−1^ min^−1^ [72] following endurance training in older adults, suggesting that combined training may be as effective for cardiorespiratory fitness improvement as traditional endurance training alone. Moreover, when compared with endurance training alone, combined training had a possibly small beneficial effect on *V*O_2peak_, thereby suggesting that combined training may be a more efficacious training strategy for improving *V*O_2peak_ in this population. Although the present investigation has not sought to understand the mechanisms of adaptation underlying training-induced changes, central and peripheral adaptations to endurance training—such as improved delivery, utilisation and extraction of oxygen—may explain increased *V*O_2peak_ [73]. In the context of combined training, it may be that the inclusion of strength training provides an additive benefit as previous investigations have reported improvements in cardiorespiratory fitness following strength training [74]—potentially mediated by increases in capillary density and mitochondrial enzyme activity [75, 76]. Furthermore, improvements in lower body strength may lead to increased time to exhaustion on an incremental exercise test, thereby increasing observed *V*O_2peak_ [77].

The effect of combined training on *V*O_2peak_ was greater for female participants, a finding in contrast to previous work, indicating that the observed response following endurance training in older adults is not moderated by sex [78]. Our findings may be related to lower cardiorespiratory fitness typically observed in females compared to males [18] as fitness improvements are typically greater for those with lower baseline fitness. For example, previous work has reported possibly small and likely moderate greater beneficial effects for participants with lower baseline fitness following endurance training and high-intensity interval training (HIT), respectively [79]. However, we observed no clear modifying effect for baseline fitness, a finding which may have been influenced by the small number and heterogeneity of studies included in our meta-regression. The complex interplay of participant (e.g., age, sex, and baseline fitness) and intervention characteristics (e.g., exercise prescription and adherence), which may influence training response, as well as between-study differences in these characteristics, also likely contributes to the observed findings. The small number of included studies means that considerable uncertainty remains, with more work needed to understand sex-specific responses to combined exercise training.

Meta-regression also demonstrated a likely small influence for shorter training programmes; a potentially meaningful finding with important practical implications as training programmes requiring a reduced time commitment may be more appealing to potential exercisers [80]. Previous work has shown that shorter duration training programmes can induce improvements in cardiorespiratory fitness that are comparable, or even greater than those following longer duration programmes [81] with gains in *V*O_2max_ similar following 8–12 or 50 weeks of training [71]. Exercise programming variables (e.g., exercise intensity, volume, and progression) represent key mediators of training response and likely play a more meaningful role than exercise programme duration in determining training adaptation [82, 83]. Between-study differences in these variables contribute to the findings reported in this meta-analysis and make it difficult to draw conclusive inference. There remains a need for future work to explore the modifying effects of programming variables on training response following combined training in older adults.

As well as cardiorespiratory fitness, maintaining muscular fitness is a primary aim of exercise training interventions in older adults because of the role which it plays in determining functional capacity with ageing [7]. Multiple methods of assessment should be used to evaluate training-induced changes in muscular fitness [84] with functional fitness tests recommended to evaluate performance in the context of the activities of daily living [85]. Typically, these measures provide a composite measure of physical capability as successful performance on these tests is determined by several components of fitness [29, 34], thereby providing a more functionally relevant and ecologically valid assessment of physical performance. The present investigation has reported small-to-moderate beneficial effects for combined training compared with no-exercise controls for 6MWT, TUG, and 30-s chair stand. These are important and clinically relevant findings as low levels of physical capability can limit older adults ability to perform the basic activities of daily living [86]. Interestingly, data also indicated a possibly small beneficial effect for combined training compared with strength training only for 30-s chair stand. However, caution is warranted in interpreting this finding as only two studies were included in the analysis.

The observed effects in this meta-analysis are greater than those reported in a previous systematic review by Baker and colleagues [87] who found that multi-modal exercise (i.e., training programmes consisting of aerobic training, strength training, and balance training) induces small and inconsistent effects on measures of functional fitness. Previous work has also demonstrated that both endurance training [88] and resistance training [9] performed in isolation are effective at eliciting positive adaptations in these measures of functional fitness. For example, Kalapotharakos and colleagues [88] evaluated the effect of a 12-week progressive high-intensity endurance training programme, performed three times per week and reported a 17% increase in 6WMT distance. This improvement was greater than the effect reported in this meta-analysis; however, the baseline fitness of the participants in this study was lower than in the present investigation. It is important to note that comparisons with previous studies are confounded by differences in participant characteristics (e.g., baseline fitness) and exercise prescription, which likely contribute to discordance between findings. More experimental work is needed to evaluate the optimal training strategy for inducing improvements in measures of functional fitness.

A range of intervention studies have documented that strength training is an effective approach for improving muscular fitness and functional performance in older adults [9, 89]. For example, lower extremity strength gain is associated with chair rise performance, gait speed, and mobility tasks [90], while strength training can improve muscle power of the lower body muscle groups relevant for carrying out daily functional tasks [91]. Several meta-analyses have extended these findings, thereby reinforcing the effectiveness of strength training for improving muscular fitness in older adults [92–94]. It, therefore, seems likely that performing strength training within a combined training programme contributes to the observed improvements reported in this investigation with these changes mediated by a range of morphological and neurological adaptations [95]. These findings have important practical implications in older adults as functional fitness is associated with reduced risk of disability and enhanced functional independence in older adults [96, 97]. In the wider context of exercise training in older adults, the observed findings are largely unsurprising based on the principle of training specificity [98, 99]. Both endurance and strength training are effective approaches for improving cardiorespiratory and muscular fitness, respectively, in older adults. Theoretically, therefore, it makes sense that the combination of these training modes would elicit improvements in both cardiorespiratory and muscular fitness.

The pooled effects presented in this meta-analysis provide an overall quantification of the effects of same-session combined training which can be used for the comparison and assessment of superiority with alternative modes of exercise training in subsequent investigations. As older adults have a clear need to maintain muscular and cardiorespiratory fitness, exercise strategies which are able to induce improvements in both of these physiological systems within the same training session may be a more efficient, and thereby, attractive proposition for potential exercisers. One potential comparator exercise mode is high-intensity interval training (HIT). Conceptually, this comparison makes sense as HIT is also capable of inducing improvements in both cardiorespiratory and muscular fitness with an exercise stimulus delivered within a single exercise session [8]. While long-term studies evaluating HIT in older adults are limited, current findings are encouraging with previous investigation demonstrating that HIT favours older and less fit individuals [79] with significant improvements in fitness possible after short-duration training programmes [8]. Future experimental work should continue to evaluate and compare the short- and long-term effects of alternate training strategies in this population to allow comprehensive evidence-based exercise recommendations to evolve.

The findings presented in the current investigation should be interpreted with caution for several reasons. First, the inclusion of only healthy community-dwelling adults aged over 50 years limits the generalisability of these results as they should not be extrapolated more widely to include frail elderly or adults with chronic long-term conditions. As considerable interindividual variation exists throughout the healthy ageing process [100], including participants characterised as non-healthy would have added further heterogeneity to this work, further limiting the impact of potential findings. However, those individuals characterised as ‘at risk’ or who are ‘non-healthy’ have potential to benefit most from therapeutic interventions such as exercise training and there remains a need for further work in these population groups. Although we defined a similar age cut-off to previous investigations [91], included studies represented a broad age range of participants. A lack of available data meant that it was not possible to perform specific analyses of typically defined discrete age categories (e.g., 50–64 years or 65–80 years), though it should be noted that there was no clear effect for age when included within our meta-regression for VO_2peak_. In addition, only one study included participants with a mean age over 80 years, an important consideration because of the projected future growth of this population group [101].

Second, several analyses presented are limited by the small numbers of eligible studies, which, combined with the between-study heterogeneity, may have affected the magnitude of observed effects and the uncertainty of these effects (e.g., the width of the reported CLs). One of the primary factors explaining the small number of included studies is the large variation in outcomes assessed and measurement tools used by different authors and research groups to evaluate training interventions. As one of the primary aims of this work was to provide practically relevant estimates of mean effects, we included only studies which contained our pre-determined specific outcome measures. In doing so, we acknowledge the exclusion of a considerable body of research. Future work is needed to fully appraise the equivalence of different measurement tools which aim to evaluate the same physical component by evaluating and comparing physical and physiological determinants of performance as well as measures of reliability, validity, and sensitivity [102]. Although challenging to implement, standardised recommendations for a battery of physical capacity tests to evaluate training interventions in older adults would also aid future attempts at synthesising research findings.

Finally, the practical implications of this work are limited by the wide variability of training programmes and incomplete reporting within the included studies. Exercise volume and intensity are both important mediators of training adaptation for both endurance and strength training [103, 104], yet the reporting of these data was inconsistent or incomplete across a number of included studies. As such, it was not possible to extract and fully evaluate the effects of exercise intensity on outcomes following combined training. While the authors of systematic reviews and meta-analyses can attempt to find further information about the study characteristics, this is a time-consuming and often ineffective process [105]. As such, the present meta-analysis is in agreement with Straight et al. [91] in calling for standardised reporting of exercise training protocols to enable researchers to fully quantify the effects of training in future meta-analyses. This should include the presentation of training programming variables (e.g., training intensity, volume, frequency, and duration) as well as information about the fidelity of the intervention [106]. While the present findings provide support for the effectiveness of same-session combined exercise training as a strategy to induce functionally relevant fitness improvements, a lack of studies including a comparator exercise mode limits the potential value of the present work and makes it difficult to draw inference about the effectiveness of this exercise approach compared with the other exercise modes typically utilised in this population (e.g., endurance or strength training). The finding that combined training improves fitness compared with no exercise is largely unsurprising and further experimental work is needed to establish superiority between exercise training modes in this population.

## Conclusions

The findings of the present meta-analysis provide further evidence supporting the application of combined exercise training as a strategy for fitness improvement in adults aged over 50 years. The quantitative mean effects presented in this investigation may help practitioners and clinicians to make informed decisions relating to future training prescription in this population. However, despite some encouraging experimental findings and the data presented in this systematic review, considerable uncertainty remains regarding the effectiveness of same-session combined exercise training compared with endurance or strength training performed alone. Further experimental work is needed to address this deficiency of knowledge and to establish superiority between these training approaches in this population. Future investigations should also seek to understand the modifying effects of exercise programming variables (e.g., volume and intensity) on training outcomes to optimise the prescription of combined training interventions.

## Electronic supplementary material

Below is the link to the electronic supplementary material.

Supplementary material 1 (PDF 266 KB)

Supplementary material 2 (PDF 282 KB)

## Data Availability

The data sets generated and analysed during the current study are available from the corresponding author on reasonable request.
