# Supplementary material for: The effects of same-session combined exercise training on cardiorespiratory and functional fitness in older adults: a systematic review and meta-analysis
Source: Aging Clin Exp Res. 2019 Jan 19;31(12):1701–17. doi: 10.1007/s40520-019-01124-7 (PMC6825647; doi:10.1007/s40520-019-01124-7)
Supplement: Supplementary file 1 — Supplementary material 1 (PDF 266 KB) [file 40520_2019_1124_MOESM1_ESM.pdf]

Name: Electronic Supplementary Material 1: Intervention characteristics of included studies

Article Title: The effects of same–session combined exercise training on cardiorespiratory and functional fitness in older adults: A systematic review and meta–analysis

Journal: Aging Clinical and Experimental Research

Authors: Christopher Hurst[1,2,3], Kathryn L Weston[4], Shaun J. McLaren[5,6] & Matthew Weston[4]

Affiliations:

[1]AGE Research Group, Institute of Neuroscience, Newcastle University, Newcastle upon Tyne, UK

[2]NIHR Newcastle Biomedical Research Centre, Newcastle upon Tyne Hospitals NHS Foundation Trust and Newcastle University, Newcastle upon Tyne, UK

[3]Newcastle University Institute for Ageing, Newcastle upon Tyne, UK

[4]School of Health and Social Care, Teesside University, Middlesbrough, UK

[5]Institute for Sport, Physical Activity and Leisure, Leeds Beckett University, Leeds, UK.

[6]The Rugby Football League, Leeds, UK

Corresponding Author: Christopher Hurst

Christopher.hurst@ncl.ac.uk

Table 1. Intervention characteristics of included studies

					Exercise prescription				Exercise order (END or STR performed first)	Other intervention components (e.g. flexibility)
					Endurance		Strength			
Study	Experimental group	Intervention duration (weeks)	Training frequency (per week)	Total Session duration (minutes)	Description of activity	Duration (minutes)	Description of activity	Duration (minutes)		
Cadore et al. (2010) [38]	COM	12	3	70	During the first 2 weeks, subjects cycled for 20 min at 80 % of HRVT2, progressing to six 4 min bouts at 100 % of HRVT (weeks 11 – 12), with 1 min of active recovery between bouts.	30	9 exercises (inclined leg–press, knee extension, leg curl, bench press, lat pull down, seated row, triceps curl, biceps curl and abdominal exercises) 3 times a week. During weeks 1 – 7, subjects performed 2 sets of 18 – 20 RM in week, progressing to 12 – 14RM (week 5). In weeks 8 – 12, subjects performed 3 sets of 12 – 14RM (week 8), advancing to 6 – 8 RM.	40	END	–
	STR	12	3	40	–	–	9 exercises (inclined leg–press, knee extension, leg curl, bench press, lat pull down, seated row, triceps curl, biceps curl and abdominal exercises) 3 times a week. During weeks 1 – 7, subjects performed 2 sets of 18 – 20 RM in week, progressing to 12 – 14RM (week 5). In weeks 8 – 12, subjects performed 3 sets of 12 – 14RM (week 8), advancing to 6 – 8 RM.	40	–	–
	END	12	3	30	During the first 2 weeks, subjects cycled for 20 min at 80 % of HRVT2, progressing to six 4 min bouts at 100 % of HR VT (weeks 11 – 12), with 1 min of active recovery between bouts.		–	–	–	–
Campos et al. (2013) [37]	COM	12	3	?	Treadmill walking. Progressing from 20 minutes @ 65% HR_{max} to 30 minutes @ 85% HR_{max}.	20–30	Eight exercises (chest press, pull down, knee extension, knee flexion, biceps pulley, triceps pulley, leg press, abdominal curl) progressing from 3 x 18–20 RM to 3 x 4–6RM.	?	END	–
	COM	12	3	?	Treadmill walking. Progressing from 20 minutes @ 65% HR_{max} to 30 minutes @ 85% HR_{max}.	20–30	Eight exercises (chest press, pull down, knee extension, knee flexion, biceps pulley, triceps pulley, leg press, abdominal curl) progressing from 3 x 18–20 RM to 3 x 4–6RM.	?	STR	–
	END	12	3	30	Treadmill walking. Progressing from 20 minutes @ 65% HR_{max} to 30 minutes @ 85% HR_{max}.	20–30	–	–	–	–
	STR	12	3	?	–	–	Eight exercises (chest press, pull down, knee extension, knee flexion,	?	–	–

							biceps pulley, triceps pulley, leg press, abdominal curl) progressing from 3 x 18–20 RM to 3 x 4–6RM.			
	CON	–	–	–	–	–	–	–	–	–
Carvalho et al. (2009) [45]	COM	35	2	60	Walking, jogging, dance, aerobics and step choreographies @ RPE of 12–14.	20–25	Muscular endurance exercises involving stair stepping, knee flexion, arm raise, shoulder abduction, shoulder adduction, shoulder rotation, squatting, biceps curl, triceps extension, toe raise, modified push up, abdominal crunch and hip extension. Exercises performed using elastic bands and free weights. 1 x 8 reps @ 12–13 RPE progressing to 3 x 15 @ 14–16 RPE.	~20	END	Agility and reaction time exercises
	CON	–	–	–	–	–	–	–	–	–
Cress et al. (1991) [56]	COM	50	3	60	Stair walking and endurance dance @ 60–75% HRR.	20	Weighted stair climbing, wall push ups, calisthenics.	?	?	–
	CON	–	–	–	–	–	–	–	–	–
Cress et al. (1999) [59]	COM	26	3	60	Kayak and single stair stepper equipment performed @ 75–80% HRR.	20	Leg press, free weights and stairmaster graviton for the upper body. Intensity was 75–80% of estimated 1RM.	20	?	–
	CON	–	–	–	–	–	–	–	–	–
Delecluse et al. (2004) [40]	COM	20	2.5	?	Cycling and walking @ 60% HRR (12-min) progressing to 80% HRR (20-min)	12-20	10 exercises (leg press, leg extension, leg curl, adductor, abductor, vertical row, chest press, arm curl, shoulder press and abdomen). 2 x 20 RM progressing to 2 x 8RM	?	END	–
	COM	20	2.5	?	Cycling and walking @ 60% HRR (12-min) progressing to 80% HRR (20-min)	12-20	10 exercises (leg press, leg extension, leg curl, adductor, abductor, vertical row, chest press, arm curl, shoulder press and abdomen). 2 x 30 RM progressing	?	END	–
	END	20	2.5	?	Cycling and walking @ 60% HRR (12-min) progressing to 80% HRR (20-min) + 12-15 min (cycling, stepping, walking) @ 65-75% HRR.	36-70	–	–	–	–
	CON	–	–	–	–	–	–	–	–	–
Desjardins–Crépeau et al. (2016) [68]	COM	12	2	60	Walking on a treadmill to maintain a moderate–high level of effort on the Borg scale. Intensity was progressed by increasing the slope of the treadmill.	30	Exercise performed using resistance cables targeting multiple lower body muscle groups. Resistance of the cables was increased as the intervention progressed.	15	STR	–

	CON	–	–	–	–	–	–	–	–	–
Douda et al. (2015) [36]	COM	39	3	45	Floor aerobic exercise, progressing to step training @ an intensity of 60–75% HR_{max}	15	3 exercises for upper– and 3 exercises for the lower–body using body weight, gym sticks and dumbbells.	15	END	–
	END	39	3	45	Floor aerobic exercise, progressing to step training @ an intensity of 60–75% HR_{max}	25	–	–	–	–
	STR	39	3	45	–	–	Exercises performed with body weight, exercise bands, gym sticks and dumbbells. Six exercises were performed for the upper body and 4 exercises for the lower body; 2–3 sets of 10–15 repetitions	30	–	–
	CON	–	–	–	–	–	–	–	–	–
Engels et al. (1998) [47]	COM	10	3	60	Aerobic dance (50–70%HR_{max})	15–30	Body weight assisted floor and chair exercises combined with elastic bands.	10–15	END	Flexibility and balance exercises (10–15 min)
	COM	10	3	60	Aerobic dance (50–70%HR_{max})	15–30	Body weight assisted floor and chair exercises combined with elastic bands.	10–15	END	Flexibility and balance exercises (10–15 min)
	CON	–	–	–	–	–	–	–	–	–
Ferketich et al. (1998) [39]	COM	12	3	~65	30 minutes @ 70% VO_{2peak} on a cycle ergometer	30	2 x 10–15 repetitions at an initial intensity of 80% of 10RM (workload increased throughout the programme). Exercises were: leg extension, calf raise, leg press, leg curl, biceps curl, triceps press, lat pull down and bench press.	~15 minutes	END	–
	END	12	3	50	30 minutes @ 70% VO_{2peak} on a cycle ergometer	30	–	–	–	–
	CON	–	–	–	–	–	–	–	–	–
García–Pinillos et al. (2017) [70]	COM	12	3	~35–40	Walking and running performed on a 400m outdoor track.	?	Two sets of: sit to stand, medicine ball chest/overhead throws, farmer walk, resistance band shoulder press, resistance band row, medicine ball squat to throw, twisting medicine ball pass.	?	?	–
	CON	–	–	–	–	–	–	–	–	–
Kim et al. (2018) [41]	COM	6	3	90	Sky walk (2 x 5 min) and Cross–country (2 x 5 min). RPE progressing from 6 (moderate) to 8 (vigorous) on the Borg scale.	20	1–3 x 12–15 reps of leg extension, pull weight and chair pull. RPE progressing from 6 (moderate) to 8 (vigorous) on the Borg scale.	50	STR	–
	STR	6	3	70	–	–	1–3 x 12–15 reps of leg extension, pull weight and chair pull.	50	–	–

							RPE progressing from 6 (moderate) to 8 (vigorous) on the Borg scale.			
	CON	–	–	–	–	–	–	–	–	–
King et al. (2002) [42]	COM	13	3	75	Brisk walking at self–selected pace of moderate intensity (RPE 12–14).	15	Combination of body–weight exercises (push–ups, dips, heel ups) and flexible closure weights (hip abduction, flexion and extension) based on a weight that could be lifted 8–10 times. Loads were increased throughout the programme.	60	END	–
	CON	–	–	–	–	–	–	–	–	–
Kwon et al. (2008) [62]	COM	24	3	60	Speedy walking, V step, Cha cha cha, Mambo step, step box aerobics, gait training with dance. Intensity progressing from 40–75% HRR	30	3–10 repetitions of push–up, modified sit–up, side leg raise, modified jathara parevartanasana, bridging of pelvis.	30	END	Balance exercises
	CON	–	–	–	–	–	–	–	–	–
Marques et al. (2009) [67]	COM	35	2	60	Walking, jogging, dancing, aerobics and step choreographies	?	Muscular endurance exercises using elastic bands and free weights.		END	Balance training
	STR	35	2	60	–	–	2 x 10–12 reps @ 60–70% 1RM. Exercises performed using variable resistance machines. Exercises were: leg press, leg extension, seated leg curl, chest press, lateral raise, overhead raise, abdominal machine.	30–40	–	–
Marques et al. (2011) [65]	COM	32	2	60	Moderate to high impact activities such as marching in place, stepping exercise and heel drops.	15	Muscular endurance activities: Squats wearing weight vest, hip flexors, extensors and abductors; knee flexors and extensors and upper–body exercises performed using elastic bands and dumbbells.	10	END	Balance (10–min) and agility training (10–min)
	CON	–	–	–	–	–	–	–	–	–
Park et al. (2008) [60]	COM	48	3	60	Weight bearing exercise at an intensity ranging from 65–70% HR_{max}.	23	Strength training	10	STR	Stretching (9–min) Balance and posture (18–min)
	CON	–	–							
Park et al. (2010) [61]	COM	12	3	90	Walking. Weeks 1–6 @ RPE ~10–11, Weeks 7–12 @ RPE ~12–13.	40	Weeks 1–6: 1 x 8–11 reps. Weeks 7–12: 1 x 12–15 reps. All exercises performed using Thera–band (green). Chest press, seated rows, shoulder press, bicep curl, tricep extension, trunk extension, abdominal crunch, squats, leg press, calf raise.	15	END	Yoga (15–min)

	CON	–	–	–	–	–	–	–	–	–
Park et al. (2015) [63]	COM	12	3	90	Running. Weeks 1–6 @ 40–55% HRR, Weeks 7–12 @ 56–75% HRR.	40	Weeks 1–6: 3 x 8–10 reps @ 60% 1RM. Weeks 7–12: 3 x 10–12 reps @ 70% 1RM. Exercises were: Arm curl, leg curl, leg extension, squat, chest press, shoulder press, lat pulldown, back extension, sit up.	30	END	–
	CON	–	–	–	–	–	–	–	–	–
Puggaard (2003) [57]	COM	35	1	60	Walking @ 69% HR_{max}	?	Rubber band or bodyweight exercises with intensity based on participant's perception.	?	?	Flexibility, reaction and balance.
	COM	35	2	60	Walking @ 62% HR_{max}	?	Rubber band or bodyweight exercises with intensity based on participant's perception.	?	?	Flexibility, reaction and balance.
	COM	35	1	60	Walking @ 72% HR_{max}	?	Rubber band or bodyweight exercises with intensity based on participant's perception.	?	?	Flexibility, reaction and balance.
	CON	–	–	–	–	–	–	–	–	–
	CON	–	–	–	–	–	–	–	–	–
	CON	–	–	–	–	–	–	–	–	–
Rubenstein et al. (2000) [66]	COM	12	3	90	Cycle, 5–15 min @ 30–80W; Treadmill, 5 min; Indoor walking 5–15 min.	15–35 min	1–3 sets of 12 repetitions at progressive intensity using ankle weights, wrist weights, elastic bands. Exercises involved: Hip flexion, hip extension, hip abduction, hip adduction, knee flexion, knee extension, squats, dorsiflexion, Plantar flexion	?	STR	Balance training (5–15 min)
	CON	–	–	–	–	–	–	–	–	–
Schaun et al. (2011) [64]	COM	12	3	35	Cycling @ 65–80% HRR.	20	Chest press, shoulder press, arm curl, lat pull down, leg press, knee extension and leg curl @ 15RM progressing to 8RM.	~15	END	–
	END	12	3	30	30 min @ 65% HRR.	30	–	–	–	–
Stewart et al. (2005) [21]	COM	26	3	?	Endurance training lasted 45 mins (treadmill, stationary cycle of stairstepper) and participants exercised at 60–90% HR_{max} with workload increased as fitness improved.	45	2 sets of 10–15 repetitions per exercise at 50% of 1RM. Exercises were lat pull down, leg extension, leg curl, bench press, leg press, shoulder press and seated row. Weight was increased when the participant could complete 15 repetitions with little difficulty.	?	STR	–
	CON	–	–							

Timmons et al. (2018) [69]	COM	12	3	40	Exercise performed using cross trainer and cycle ergometer. 3 x 4 min exercise @ power output to elicit a target intensity of 80% of age–predicted maximum heart rate (%HR_{max}).	12	Training consisted of six exercise machines (leg press, seated row, chest press, lat pulldown, leg extension, and tricep dips). 15 tempo–controlled repetitions of one exercise in a 60 sec period followed by 30 sec of rest before the next exercise. 2 x 6 exercises were completed in each session. The intervention began at ~60% of 1RM, but once an exercise could be completed comfortably for the 60 sec period, an ~5% increment in weight to be lifted was added for the next training session.	12	Merged (STR, END, STR, END)	–
	END	12	3	40	Exercise performed using cross trainer and cycle ergometer. 6 x 4 min exercise @ power output to elicit a target intensity of 80% of age–predicted maximum heart rate (%HR_{max}).	24	–	–	–	–
	STR	12	3	40	–	–	Training consisted of six exercise machines (leg press, seated row, chest press, lat pulldown, leg extension, and tricep dips). 15 tempo–controlled repetitions of one exercise in a 60 sec period followed by 30 sec of rest before the next exercise. 4 x 6 exercises were completed in each session. The intervention began at ~60% of 1RM, but once an exercise could be completed comfortably for the 60 sec period, an ~5% increment in weight to be lifted was added for the next training session.	24	–	–
	CON	–	–	–	–	–	–	–	–	–
Villareal et al. (2011) [58]	COM	52	3	90	Walking on a treadmill, stationary cycling and stair climbing (65% HR_{peak} increasing to 70–85% HR_{peak}).	?	1 or 2 x 8 x 12 reps @ ~65% 1RM increasing to 2–3 x 6–8 reps @ ~80% 1RM. Exercises were nine upper– and lower–extremity exercises using weight machines.	?	END	Flexibility and balance
	CON	–	–		–	–	–	–	–	–
Wang et al. (2015) [44]	COM	12	3	60	Stepping, marching, brisk walking, and spinning a hula hoop with upper limb movements were included in the endurance exercise. The	20	The resistance training targeted the lower extremity muscles and involved hip flexion, hip extension, knee flexion, ankle plantarflexion, bodyweight squats in standing, knee ex– tension in sitting, and ankle dorsiflexion in supine.	20	STR	Balance training (20–min)

					training intensity was set at 70–75% HR_{max}.		Intensity began from 50 % maximal voluntary contraction for ten repetitions and then progressively increased to 75–80 % maximal voluntary contraction.			
	CON	–	–	–	–	–	–	–	–	–
Wilhelm et al. (2014) [46]	COM	12	2	?	Continuous load upright cycling (pedaling cadence 80–90 rpm) progressing from 20–min @ 85% HRVT2 to 40–min @ 95% HRVT2.	20–40	Major muscle groups were exercised by the following exercises performed with either machines or dumbbells progressing from 2 x 15–18RM to 3 x 8–10RM): lat pull down, bench press, elbow extension, and dumbbell bicep curls for the upper–body; and leg press, knee extension, and knee flexion for the lower–body.	?	STR	–
	COM	12	2	?	Continuous load upright cycling (pedaling cadence 80–90 rpm) progressing from 20–min @ 85% HRVT2 to 40–min @ 95% HRVT2.	20–40	Major muscle groups were exercised by the following exercises performed with either machines or dumbbells progressing from 2 x 15–18RM to 3 x 8–10RM): lat pull down, bench press, elbow extension, and dumbbell bicep curls for the upper–body; and leg press, knee extension, and knee flexion for the lower–body.	?	END	–
	CON	–	–	–	–	–	–	–	–	–

Abbreviations: *COM*, combined training; *END*, endurance training only; *STR*, strength training only; *CON*, no–exercise control group; *RM*, repetition maximum; *RPE*, rating of perceived exertion; *HRR*, heart rate reserve; *HRVT2*, heart rate equivalent to the second ventilatory threshold; *?*, Data unknown / not presented by authors; –, N/A
