# Supplementary material for: The effects of same-session combined exercise training on cardiorespiratory and functional fitness in older adults: a systematic review and meta-analysis
Source: Aging Clin Exp Res. 2019 Jan 19;31(12):1701–17. doi: 10.1007/s40520-019-01124-7 (PMC6825647; doi:10.1007/s40520-019-01124-7)
Supplement: Supplementary file 2 — Supplementary material 2 (PDF 282 KB) [file 40520_2019_1124_MOESM2_ESM.pdf]

Name: Electronic Supplementary Material 2: Risk of bias assessment

Article Title: The effects of same-session combined exercise training on cardiorespiratory and functional fitness in older adults: A systematic review and meta-analysis

Journal: Aging Clinical and Experimental Research

Authors: Christopher Hurst[1,2,3], Kathryn L Weston[4], Shaun J. McLaren[5,6] & Matthew Weston[4]

Affiliations:

[1]AGE Research Group, Institute of Neuroscience, Newcastle University, Newcastle upon Tyne, UK

[2]NIHR Newcastle Biomedical Research Centre, Newcastle upon Tyne Hospitals NHS Foundation Trust and Newcastle University, Newcastle upon Tyne, UK

[3]Newcastle University Institute for Ageing, Newcastle upon Tyne, UK

[4]School of Health and Social Care, Teesside University, Middlesbrough, UK

[5]Institute for Sport, Physical Activity and Leisure, Leeds Beckett University, Leeds, UK.

[6]The Rugby Football League, Leeds, UK

Corresponding Author: Christopher Hurst

Christopher.hurst@ncl.ac.uk

	Random sequence generation (selection bias)	Allocation concealment (selection bias)	Blinding of participants and personnel (performance bias)	Blinding of outcome assessment (detection bias)	Incomplete outcome data (attrition bias)	Selective reporting (reporting bias)	Other bias
Cadore et al. (2010)	?	?	+	?	+	?	+
Campos et al. (2013)	?	?	+	?	+	?	+
Carvalho et al. (2009)	?	?	+	?	+	?	+
Cress et al. (1991)	−	−	+	?	+	?	+
Cress et al. (1999)	?	?	+	?	+	?	+
Delecluse et al. (2004)	?	?	+	?	+	?	+
Desjardins-Crepeau et al. (2016)	+	?	+	+	+	?	+
Douda et al. (2015)	−	−	+	?	?	?	+
Engels et al. (1998)	?	?	+	?	+	?	+
Ferketich et al. (1998)	?	?	+	?	+	?	+
Garcia-Pinillos et al. (2017)	?	?	+	?	+	?	+
Kim et al. (2018)	+	?	+	?	+	?	+
King et al. (2002)	+	?	+	+	+	?	+
Kwon et al. (2008)	−	−	+	?	?	?	+
Marques et al. (2009)	?	?	+	+	+	?	+
Marques et al. (2011)	+	+	+	?	+	?	+
Park et al. (2008)	+	?	+	?	+	?	+
Park et al. (2010)	?	?	+	?	?	?	+
Park et al. (2015)	?	?	+	?	+	?	+
Puggaard et al. (2003)	?	?	+	?	+	?	+
Rubenstein et al. (2000)	+	?	+	?	+	?	+
Schaun et al. (2011)	?	?	+	?	+	?	+
Stewart et al. (2005)	?	?	+	?	+	?	+
Timmons et al. (2018)	+	+	+	?	+	?	+
Villareal et al. (2011)	?	?	+	?	+	?	+
Wang et al. (2015)	−	−	+	?	+	?	+
Wilhelm et al. (2014)	−	−	+	?	+	?	+
